# Duodenal Tumor Presenting With Persistent Vomiting: Diagnostic and Therapeutic Challenges

**DOI:** 10.7759/cureus.99506

**Published:** 2025-12-17

**Authors:** Aleksandra Tlak, Katarzyna Bielawska, Wiktoria J Auguścik

**Affiliations:** 1 Internal Medicine, Grochowski Hospital, Warsaw, POL; 2 Internal Medicine, District Health Centre, Otwock, POL

**Keywords:** bowel obstruction, diagnostic challenges, duodenal tumor, enteral nutrition, persistent vomiting

## Abstract

Duodenal tumors are rare neoplasms of the small intestine, often diagnosed at an advanced stage due to nonspecific clinical presentation and diagnostic challenges. We present the case of a 40-year-old patient hospitalized for vomiting, abdominal pain, and unintentional weight loss. During hospitalization, a duodenal mass was diagnosed, along with multiple liver metastases and associated gastrointestinal obstruction. This report discusses the epidemiology, pathogenesis, classification, clinical presentation, diagnostic methods, and therapeutic strategies for duodenal tumors, with particular emphasis on progressive intestinal obstruction and the need for appropriate nutritional interventions. This case highlights the importance of early diagnosis and an interdisciplinary approach in managing this rare disease.

## Introduction

Duodenal tumors are rare neoplasms of the small intestine, accounting for less than one percent of all gastrointestinal malignancies [1-4]. They predominantly affect middle-aged and elderly individuals, with a slight male predominance. Histologically, duodenal tumors constitute a diverse group comprising several categories: epithelial neoplasms (adenomas and adenocarcinomas), neuroendocrine tumors, mesenchymal tumors such as gastrointestinal stromal tumors, and lymphomas. Epidemiological studies indicate that adenocarcinoma constitutes the majority of duodenal tumors, and their incidence is higher in populations with chronic gastrointestinal diseases and genetic syndromes predisposing to small bowel cancer [3]. Tumors may arise in the superior, descending, transverse, or ascending duodenum, often leading to severe complications, including intestinal obstruction [4]. The rarity of the disease and its nonspecific clinical symptoms frequently result in delayed diagnosis at advanced stages [5]. A delayed diagnosis means that duodenal tumors are often detected at an advanced stage, in which the large tumor mass may cause progressive gastrointestinal obstruction, leading to a number of serious clinical complications and, in some cases, indicating the need to initiate appropriate nutritional interventions.

## Case presentation

A 40-year-old male patient in moderate general condition was admitted to the Department of Internal Medicine with a history of vomiting persisting for one week, right-sided abdominal pain, and diarrhea. Additionally, the patient reported an unintentional weight loss in relation to the current symptoms. His past medical history included arterial hypertension treated with perindopril, amlodipine, and indapamide. The patient denied addictions and allergies. There were no significant findings in his past medical history or the medical history of his family. On physical examination, signs of dehydration (dry mucous membranes) were noted, as well as hypotension (65/40 mmHg), tenderness in the right lower quadrant of the abdomen without palpable masses, abdominal distension above the thoracic level, and audible peristalsis. Laboratory tests showed dehydration-related kidney injury, as well as metabolic alkalosis associated with severe vomiting (the development of the metabolic alkalosis may also have been influenced by the patient’s chronic use of diuretic medications). Tests also showed elevated inflammatory markers, hyponatremia, hypocalcemia, and elevated cholestasis markers (Table 1).

**Table 1:** Laboratory tests results WBC: White Blood Cell count; CRP: C-Reactive Protein; pH: arterial blood pH; HCO₃: bicarbonate; eGFR: estimated Glomerular Filtration Rate; Sodium: Na⁺; Calcium: Ca²⁺; GGT: Gamma-Glutamyl Transferase

Parameter	WBC	CRP	pH	HCO3-	eGFR	Sodium	Calcium	GGT
Patient result	19.95 × 10³/µL	98.9 mg/L	7.461	34.7 mmol/L	4.46 ml/min/1.73 m²	129 mmol/L	1.95 mmol/L	155.1 U/L
Reference range	4.0–11.0 × 10³/µL	<5 mg/L	7.35–7.45	22–26 mmol/L	>90 ml/min/1.73 m²	135–145 mmol/L	2.25–2.75 mmol/L	< 40 U/L

**Figure 1:**
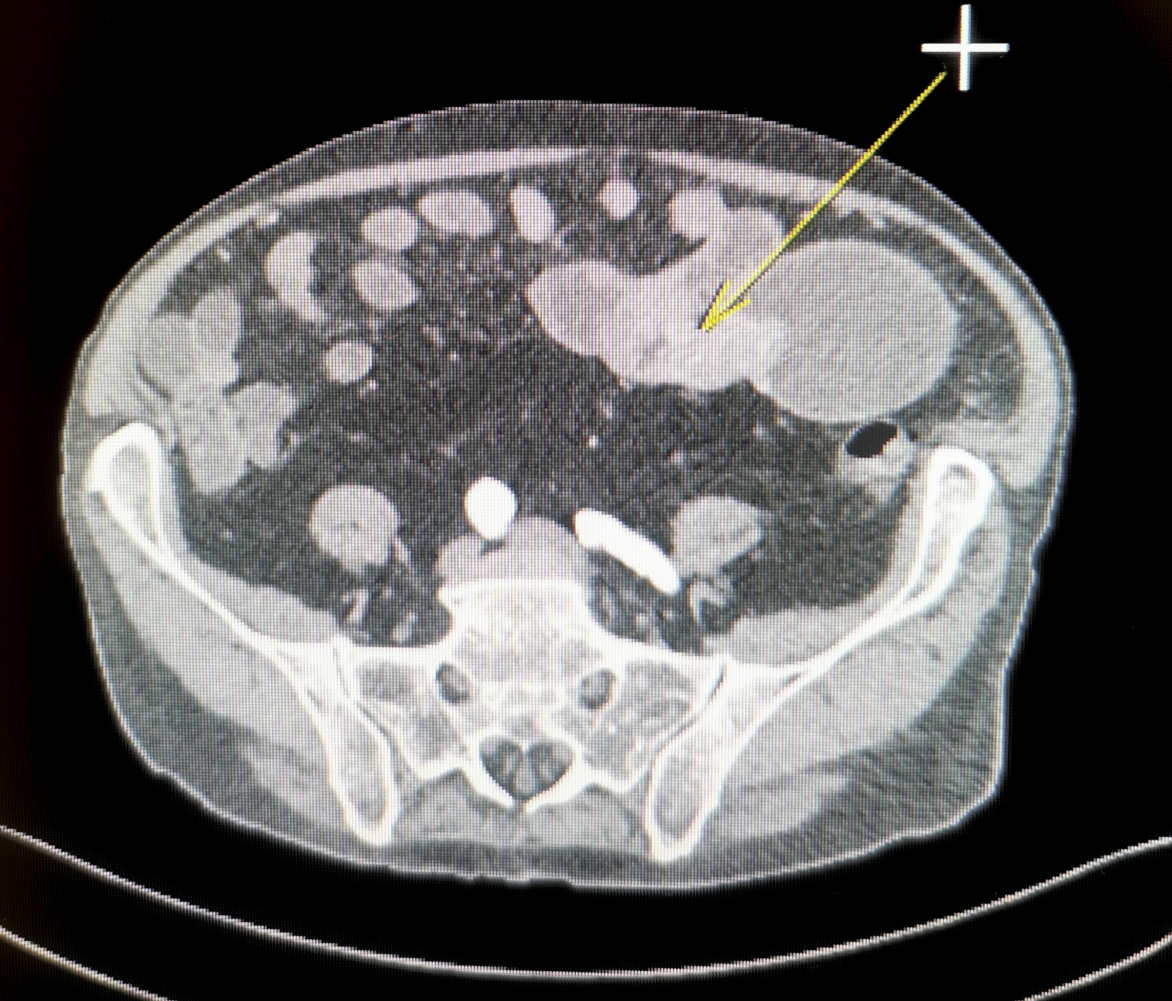
Duodenum with features of disseminated malignancy—yellow arrow. Dilatation of the horizontal duodenal segment with an intramural mass (25×30×25 mm) at the duodenojejunal junction, causing luminal narrowing

**Figure 2:**
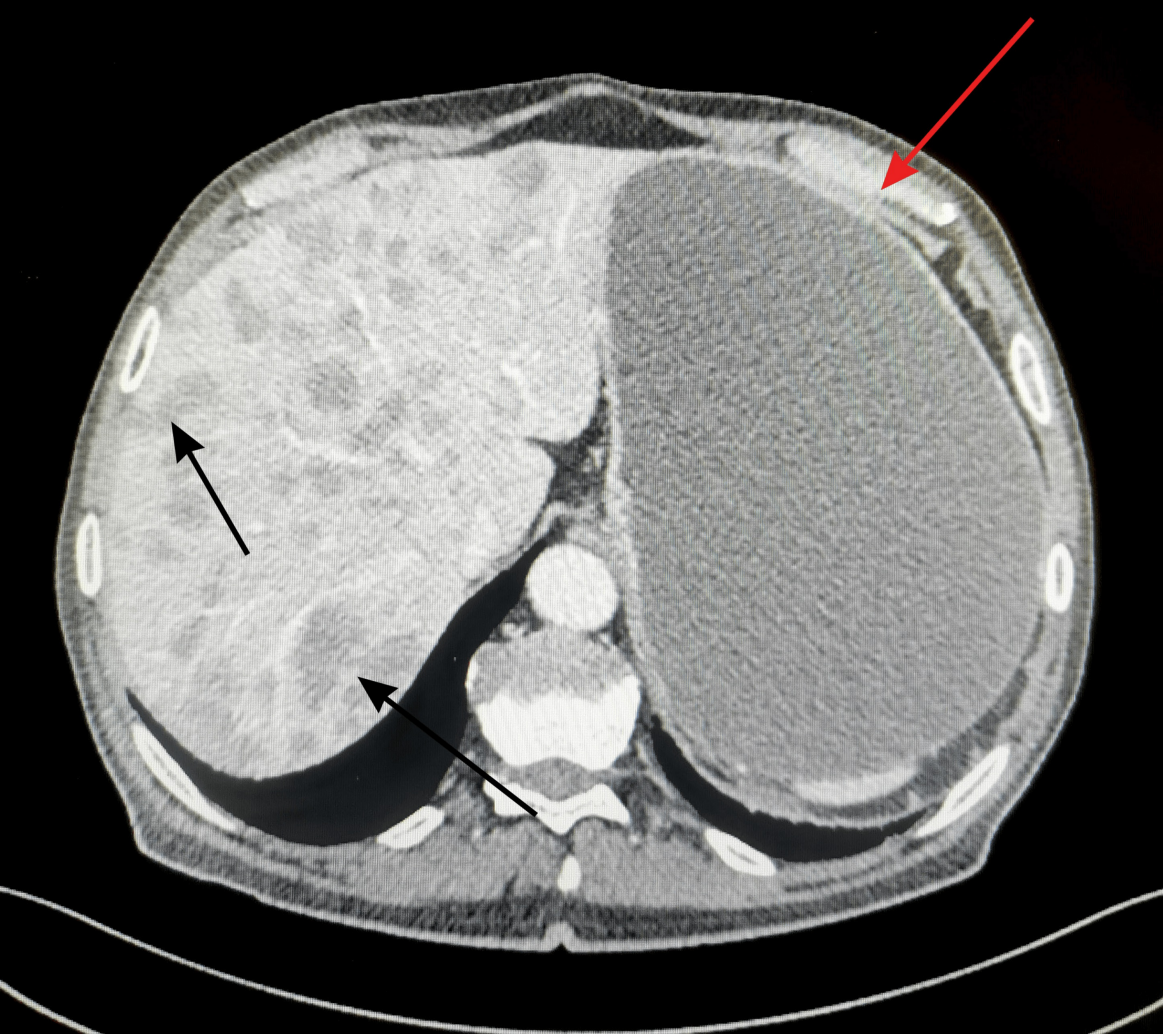
A large amount of retained gastric content (20×22×15 cm)—red arrow. Numerous hepatic lesions up to 29 mm, consistent with metastases—black arrows

After fluid resuscitation and stabilization of vital signs, imaging and endoscopic diagnostics were performed. High-resolution CT of the abdomen revealed the following lesions (Figures 1, 2).

Endoscopy demonstrated, approximately 20 cm distal to the papilla of Vater, a polycyclic mass protruding into the lumen, obstructing the passage of food. Residual gastric content was present proximal to the lesion. Biopsies were taken for histopathological analysis. Based on these findings, disseminated malignant disease was suspected: a tumor of the descending duodenum, with concurrent intestinal obstruction. Histopathological examination revealed a tubulovillous adenoma with malignant transformation.

After surgical consultation, the patient was qualified for tumor resection with gastrojejunostomy. During subsequent hospitalization, symptoms of high intestinal obstruction worsened, and an attempted endoscopic placement of a nasojejunal tube for enteral feeding was unsuccessful. Consequently, the patient qualified for jejunostomy.

During surgery, resection of the duodenal tumor was performed; however, implantation of a jejunostomy was abandoned due to progressive deterioration of the patient’s general condition intraoperatively. Due to signs of cardiorespiratory failure, the patient was immediately transferred to the Intensive Care Unit. In the Intensive Care Unit, the patient was placed on mechanical ventilation, high-dose norepinephrine infusion was initiated, and antibiotic therapy was administered. Laboratory tests during hospitalization revealed features of renal failure, persistently elevated inflammatory markers, and positive bacterial blood cultures unresponsive to antibiotic treatment. Over the subsequent days, despite intensive therapy, the patient remained in an extremely critical general condition with progressive multiorgan failure. After two weeks of hospitalization in the Intensive Care Unit, the patient was pronounced dead.

## Discussion

The pathogenesis of duodenal tumors is complex and includes both genetic and environmental factors (radiation, certain chemical compounds, and infections). Significant risk factors comprise polyposis syndromes such as familial adenomatous polyposis (FAP), Lynch syndrome, Peutz-Jeghers syndrome, chronic inflammatory conditions of the duodenum and small intestine, smoking, and diets rich in red meat and sugars [3-4]. Histopathologically, duodenal tumors represent a heterogeneous group, including epithelial neoplasms (adenomas and adenocarcinomas), neuroendocrine tumors (NETs) of varying malignancy, mesenchymal tumors such as gastrointestinal stromal tumors (GISTs), and lymphomas [6]. The most common histological type in this location is adenocarcinoma [4]. The pathogenesis of adenocarcinoma follows the adenoma-carcinoma sequence [5]. Various mutations initiate epithelial or neuroendocrine proliferation, followed by submucosal invasion and dissemination to lymph nodes and the liver [4].

Clinically, duodenal tumors present with nonspecific symptoms such as abdominal pain, vomiting, diarrhea, weight loss, dehydration, and gastrointestinal bleeding, and general cancer-related symptoms (fever, night sweats, and weakness). Periampullary lesions may cause obstructive jaundice [4]. In distal duodenal tumors, as in the present case, progressive obstruction may develop, manifesting as persistent vomiting, constipation, inability to pass gas, increased intragastric pressure, abdominal pain, and a feeling of fullness in the upper abdomen. Progressive obstruction with associated vomiting and dehydration may lead to electrolyte disturbances (such as hyponatremia and hypokalemia), metabolic alkalosis, and renal failure.

The gold standard in diagnosing duodenal tumors remains gastroduodenoscopy with biopsy [4,7]. High-resolution CT and MRI are widely used to evaluate luminal narrowing, tumor extent, local invasion, and metastatic spread [3,4]. In the present case, HRCT revealed multiple hepatic metastases and features of intestinal obstruction.

Surgical resection remains the mainstay of treatment for duodenal tumors [1,8]. Systemic therapies, such as chemotherapy and targeted treatments, may be considered in advanced neuroendocrine tumors or adenocarcinomas [2,8-9]. Each therapeutic decision should be tailored to the individual patient. Progressive obstruction is a frequent complication and requires prompt surgical or endoscopic intervention [10].

Restoring intestinal continuity and ensuring adequate nutrition are critical for prognosis and the continuation of oncological therapy. Enteral nutrition after resection of duodenal tumors supports normal physiological intestinal function, reduces the risk of sepsis and metabolic complications, and improves the postoperative immune response, which may contribute to a shorter hospital stay. Parenteral nutrition is used when enteral feeding is not feasible, for example, in patients with persistent gastrointestinal obstruction after surgery. This type of nutrition allows precise dosing of nutrients but carries an increased risk of infections, metabolic complications, and intestinal atrophy. Therefore, enteral nutrition is the preferred method for feeding patients with duodenal tumors, while parenteral nutrition serves as an alternative in critical situations [10].

## Conclusions

Although rare, duodenal tumors can cause severe complications, including gastrointestinal obstruction. Their nonspecific symptoms often delay diagnosis. In the diagnostic workup, assessment of a potential genetic basis for tumor occurrence is important, particularly in the population of young patients. Endoscopy with biopsy and computed tomography remain the gold standard of diagnostic assessment. Due to histological variability, histopathological evaluation of the specimen is crucial in post-endoscopic management. Surgical management with restoration of gastrointestinal continuity, as well as nutritional support via enteral or parenteral routes, is key to improving patient outcomes. This case underscores the importance of comprehensive and multidisciplinary care in managing patients with duodenal tumors, which can pose a very serious threat to health and, in the presented case, even to the patients’ lives.

## References

[1] Poultsides GA, Huang LC, Cameron JL (2012). Duodenal adenocarcinoma: clinicopathologic analysis and implications for treatment. Ann Surg Oncol.

[2] Meijer LL, Alberga AJ, de Bakker JK (2018). Outcomes and treatment options for duodenal adenocarcinoma: a systematic review and meta-analysis. Ann Surg Oncol.

[3] Nakagawa K, Sho M, Fujishiro M (2022). Clinical practice guidelines for duodenal cancer 2021. J Gastroenterol.

[4] Cloyd JM, George E, Visser BC (2016). Duodenal adenocarcinoma: advances in diagnosis and surgical management. World J Gastrointest Surg.

[5] Blanco-Fernández G, Aparicio-López D, Villodre C (2024). Duodenal adenocarcinoma: the relationship between type of surgery and site of recurrence in a Spanish cohort. Gastroenterology Insights.

[6] Barat M, Dohan A, Dautry R (2017). Mass-forming lesions of the duodenum: a pictorial review. Diagn Interv Imaging.

[7] Esaki M, Suzuki S, Ikehara H (2018). Endoscopic diagnosis and treatment of superficial non-ampullary duodenal tumors. World J Gastrointest Endosc.

[8] Zhang Z, Lei Y, Wang D (2022). Case report: a case of advanced duodenal adenocarcinoma in complete remission after chemotherapy combined with targeted therapy and radiotherapy. Front Oncol.

[9] Liao LG, Xiong ZG, Hu JJ (2025). Progress in the treatment of duodenal cancer: a comprehensive review. World J Gastrointest Oncol.

[10] Ibrahim R, Yassine M, Dika Z (2025). Proximal bowel obstruction caused by high-grade duodenal adenocarcinoma: a rare case. Ann Med Surg (Lond).

